# A novel end-to-end learning framework for inferring lncRNA-disease associations based on convolution neural network

**DOI:** 10.3389/fgene.2025.1580512

**Published:** 2025-04-09

**Authors:** Shunxian Zhou, Sisi Chen, Jinhai Le, Yangtai Xu, Lei Wang

**Affiliations:** ^1^ College of Information Science and Engineering, Hunan Women’s University, Changsha, China; ^2^ The First Hospital of Hunan University of Chinese Medicine, Changsha, China; ^3^ Intelligent Equipment School, Changsha Rail Transit Institute, Changsha, China; ^4^ Changsha Technology Innovation Center of Artificial Intelligence Large Model Training, Changsha University, Changsha, China

**Keywords:** lncRNA-disease associations, computational model, prediction model, convolutional neural network, negative samples

## Abstract

**Introduction:**

In recent years, lots of computational models have been proposed to infer potential lncRNA-disease associations.

**Methods:**

In this manuscript, we introduced a novel end-to-end learning framework named CNMCLDA, in which, we first adopted two convolutional neural networks to extract hidden features of diseases and lncRNAs separately. And then, by combining these hidden features of diseases and lncRNAs with known lncRNA-disease associations, we designed five different loss functions. Next, based on errors obtained by these loss functions, we would perform back propagation to fit parameters in CNMCLDA, and complete those missing values in lncRNA-disease relational matrix according to these fitted parameters. In order to demonstrate the prediction performance of CNMCLDA, intensive experiments have been carried out and experimental results show that CNMCLDA can achieve better performances than state-of-the-art competitive predictive models in frameworks of five-fold cross validation, ten-fold cross validation and leave-one-disease-out cross validation respectively.

**Results and Discussion:**

Moreover, in case studies of gastric cancer, glioma and breast cancer, there are 19, 17 and 16 out of top 20 candidate lncRNAs inferred by CNMCLDA having been confirmed by recent relevant literatures separately, which demonstrated the outstanding performance of CNMCLDA as well. Hence, it is obvious that CNMCLDA may be an effective tool for prediction of potential lncRNA-disease associations in the future.

## 1 Introduction

In the last few years, more and more researches have pointed out that lncRNAs play a significant role in some biological processes (Statello et al., 2021) and are associated with many human diseases including HIV (Zhou et al., 2023), cardiovascular diseases (Congrains et al., 2012), leukemia (Calin et al., 2007), various cancers (Yang et al., 2023; Gupta et al., 2010; Ci et al., 2024), etc. Hence, prediction of possible associations between lncRNAs and diseases can not only contribute to understand the pathogenesis of human diseases at the molecular level, but also provide a novel perspective for new drug development and personalized medication (Wu et al., 2021). Up to now, researchers have established a series of publicly available databases including lncRNAdb (Amaral et al., 2011; Cheng et al., 2015), NONCODE (Bu et al., 2012), LncRNADisease (Bao et al., 2019), and NRED (Dinger et al., 2009) etc., and based on these databases, lots of computational methods have been proposed successively, which can be roughly classified into three major categories according to their implementation strategies (Chen et al., 2017; Fan et al., 2022). The first type of approach is mainly based on different machine learning models, for instance, Zhou and Peng et al. established a prediction model by using a boosting-based ensemble learning model (Zhou et al., 2024). Yu and Wang et al. adopted the Naïve Bayes classifier to predict potential associations between lncRNAs and diseases (Jingwen et al., 2018; Yu et al., 2019). Xuan and Wang et al. utilized probability matrix decomposition to infer latent lncRNA-disease associations (Xuan et al., 2019). Wang et al. developed a novel model named gGATLDA for lncRNA-disease association prediction based on graph-level graph attention network (Wang and Zhong, 2022). Zhang et al. designed a lncRNA-disease association prediction tool development based on bridge heterogeneous information network via graph representation learning for family medicine and primary care (Zhang et al., 2023). The second type of approach is mainly based on the network topologies, for example, Sun et al. predicted potential lncRNA-disease association by applying random walk with restart on the lncRNA functional similarity network (Sun et al., 2014). Zhang et al. proposed a computational model by implementing flow propagation algorithm on multiple heterogeneous networks (Zhang et al., 2017). Chen et al. constructed an effective prediction model named KATZLDA by integrating the lncRNA functional similarity and the disease semantic similarity with known lncRNA-disease associations (Chen, 2015a). Different from the above two types of methods, which mainly rely on known lncrNa-disease associations verified by biological experiments to infer potential lncrNa-disease associations, the third type of approach mainly focuses on adopting indirect biological information to infer potential lncRNA-disease associations, which can achieve satisfactory predictive performance while lack of known lncRNA-disease associations. For example, Liu et al. established a novel computational model by combining disease genes and expression profiles of lncRNA (Liu et al., 2014). Through above descriptions, it is easy to know that those existing computational models exist the following limitations: (1) Lots of existing methods are strongly dependent on known lncRNA-disease associations. (2) Machine learning based methods randomly select unlabeled samples as negative samples, or directly take all unlabeled samples as negatives. (3) Most existing methods cannot predict potential associations between lncRNAs and diseases having no known associations with lncRNAs.

Therefore, in order to overcome above limitations of traditional forecasting models, in this paper, the prediction of potential diseases related lncRNAs will first be regarded as completion of missing values in a lncRNA-disease relational matrix, which has been demonstrated to be practical and effective in many bioinformatics fields. For example, in 2022, Yan et al. proposed a matrix completion model for drug repositioning (Yan et al., 2022), which can achieve satisfactory prediction performance. In 2024, Shi et al. designed a novel prediction model, which can effectively infer potential associations between microbes and diseases based on graph autoencoder and inductive matrix completion (Shi et al., 2024). Certainly, there are also some computational models designed to predict potential lncRNA-disease associations based on the idea of matrix completion. For instances, Lu et al. constructed a lncRNA-disease association prediction model based on the inductive matrix completion (Lu et al., 2018). Different from these existing matrix completion based models, in this paper, we developed a novel end-to-end learning framework called CNMCLDA to complete the lncRNA-disease relational matrix, in which, we combined known lncRNA-disease associations with known lncRNA-miRNA associations and known miRNA-disease associations, which ensured that CNMCLDA could achieve better performance than those existing prediction models based only on known lncRNA-disease associations. And at the same time, we further integrated five different loss functions to update parameters in CNMCLDA, and considered the balance between positive and passive samples in CNMCLDA, thus ensuring that CNMCLDA would be more powerful and effective. Finally, in order to demonstrate the effectiveness and superiority of CNMCLDA, we first compared it with nine state-of-the-art models under frameworks of 5-fold CV (cross-validation) and 10-fold CV respectively, and experimental results showed that CNMCLDA achieved reliable AUC values of 0.9235 and 0.9446 in 5-fold CV and 10-fold CV separately, which were higher than all those competitive models. Secondly, in view of limitation that some existing models cannot be applied to infer potential associations between lncRNAs and diseases without known associated lncRNAs, we further adopted a novel evaluation index named LODOCV (leave-one-disease-out cross validation) to assess the predictive performance between CNMCLDA and four of above nine state-of-the-art models that can be applied to infer potential associations between lncRNAs and diseases without known associated lncRNAs, and experimental results illustrated that CNMCLDA achieved better performance than all these competitive models simultaneously. Furthermore, in order to verify the adaptability of CNMCLDA, we downloaded and applied another different dataset to evaluate the prediction performance of CNMCLDA, and experimental results showed that CNMCLDA achieved satisfactory performance as well. Finally, in case studies of gastric cancer, glioma and breast cancer, experimental results illustrated that there were 19,17 and 16 of top 20 candidate lncRNAs predicted by CNMCLDA having been confirmed by recent literatures, which also demonstrated that CNMCLDA may become a vital tool to explore potential relationships between lncRNAs and diseases in the future.

## 2 Materials

### 2.1 Data collection and preprocessing

In this section, we firstly collected known miRNA-disease associations, miRNA-lncRNA associations and lncRNA-disease associations from public databases of HMDD (Cui et al., 2023), starBasev2.0 (Li et al., 2014) and MNDRv2.0 (Cui et al., 2018) respectively. After removing duplicated associations, we finally obtained 246 different miRNAs, 1,089 different lncRNAs, 373 different diseases, a dataset *S*_*MD*_ consisting of 4,704 known miRNA-disease associations between all these 246 miRNAs and 373 diseases, a dataset *S*_*ML*_ consisting of 9,086 known miRNA-lncRNA associations between all these 246 miRNAs and 1,089 lncRNAs, and a dataset *S*_*LD*_ consisting of 407 known lncRNA-disease associations between 77 of all these 1,089 lncRNAs and 95 of all these 373 diseases. For convenience, let *nm*, *nd*, *nl*, *nl_ld* and *nd_ld* denote the numbers of all these 246 miRNAs, 373 diseases, 1,089 lncRNAs, 77 lncRNAs and 95 diseases separately, and *NM*, *ND*, *NL*, *NL_LD* and *ND_LD* represent the sets consisting of all these 246 miRNAs, 373 diseases, 1,089 lncRNAs, 77 lncRNAs and 95 diseases respectively, then a *nm* × *nd* dimensional matrix *MD*, a *nm* × *nl* dimensional matrix *ML*, and a *nl_ld* × *nd_ld* dimensional matrix *LD* could be constructed based on above three kinds of datasets *S*_*MD*_, *S*_*ML*_ and *S*_*LD*_ respectively. Here, as for the matrix *MD*, there is *MD*(*i*, *j*) = 1, if and only if there is a known association between the given miRNA *m*_*i*_ and the given disease *d*_*j*_ in *S*_*MD*_, otherwise, there is *MD*(*i*, *j*) = 0. As for the matrix *ML*, There is *ML*(*i*, *j*) = 1, if and only if there is a known association between the given miRNA *m*_*i*_ and the given lncRNA *l*_*j*_ in *S*_*ML*_, otherwise, there is *ML*(*i*, *j*) = 0. And as for the matrix *LD*, there is *LD*(*i*, *j*) = 1, if and only if there is a known association between the given lncRNA *l*_*i*_ and the given disease *d*_*j*_ in *S*_*LD*_, otherwise, there is *LD*(*i*, *j*) = 0. The detailed information about the data downloaded from these three public databases HMDD, starBasev2.0 and MNDRv2.0 is illustrated in Table 1.

**TABLE 1:** Data downloaded from public databases HMDD, starBasev2.0 and MNDRv2.0.

Database	miRNA	Disease	lncRNA	miRNA-disease associations	miRNA-lncRNA associations	lncRNA-disease associations
HMDD	246	373		4,704		
starBase v2.0	246		1,089		9,086	
MNDR v2.0		95	77			407

### 2.2 Calculation of disease semantic similarity and lncRNA function similarity

In recent years, the semantic similarity of disease has been widely utilized in the field of bioinformatics, and especially in prediction of associations between diseases and lncRNAs (Wang et al., 2019; Xiao et al., 2020). In this section, we would adopt the semantic similarity of disease in CNMCLDA in the following way: Firstly, for each disease downloaded above, we would obtain its corresponding MESH (Medical Subject Headings) descriptors from the U.S. National Library of Medicine (http://www.nlm.nih.gov/), which was denoted as a Directed Acyclic Graph (DAG). And then, base on these DAGs, we would obtain the semantic similarity scores across all diseases, and a corresponding semantic similarity score matrix *S*_*D*_∈*R* ^*nd*×*nd*^. Next, by combining the matrix *S*_*D*_ with the matrix *LD* obtained previously, we would further adopt the method proposed in reference (Xiao et al., 2020) to calculate the functional similarity of lncRNA, and obtain a corresponding functional similarity score matrix *S*_*L*_∈*R* ^*nl*^ ^x^ ^*nl*^ as well.

## 3 Construction of the CNMCLDA

The goal of CNMCLDA is to fill those missing values in the original lncRNA-disease relational matrix *LD*. The traditional solution is to find two matrix *W* and *H* that satisfy the following Formula 1:

$$\min{\left\|LD-S_{D}WH^{T}S_{L}^{T}\right\|}_{F}^{2}+\lambda_{1}{\left\|W\right\|}_{F}^{2}+\lambda_{1}{\left\|H\right\|}_{F}^{2}\;s.t.\;W\geq 0,H\geq 0$$ (1)

**FIGURE 1:**
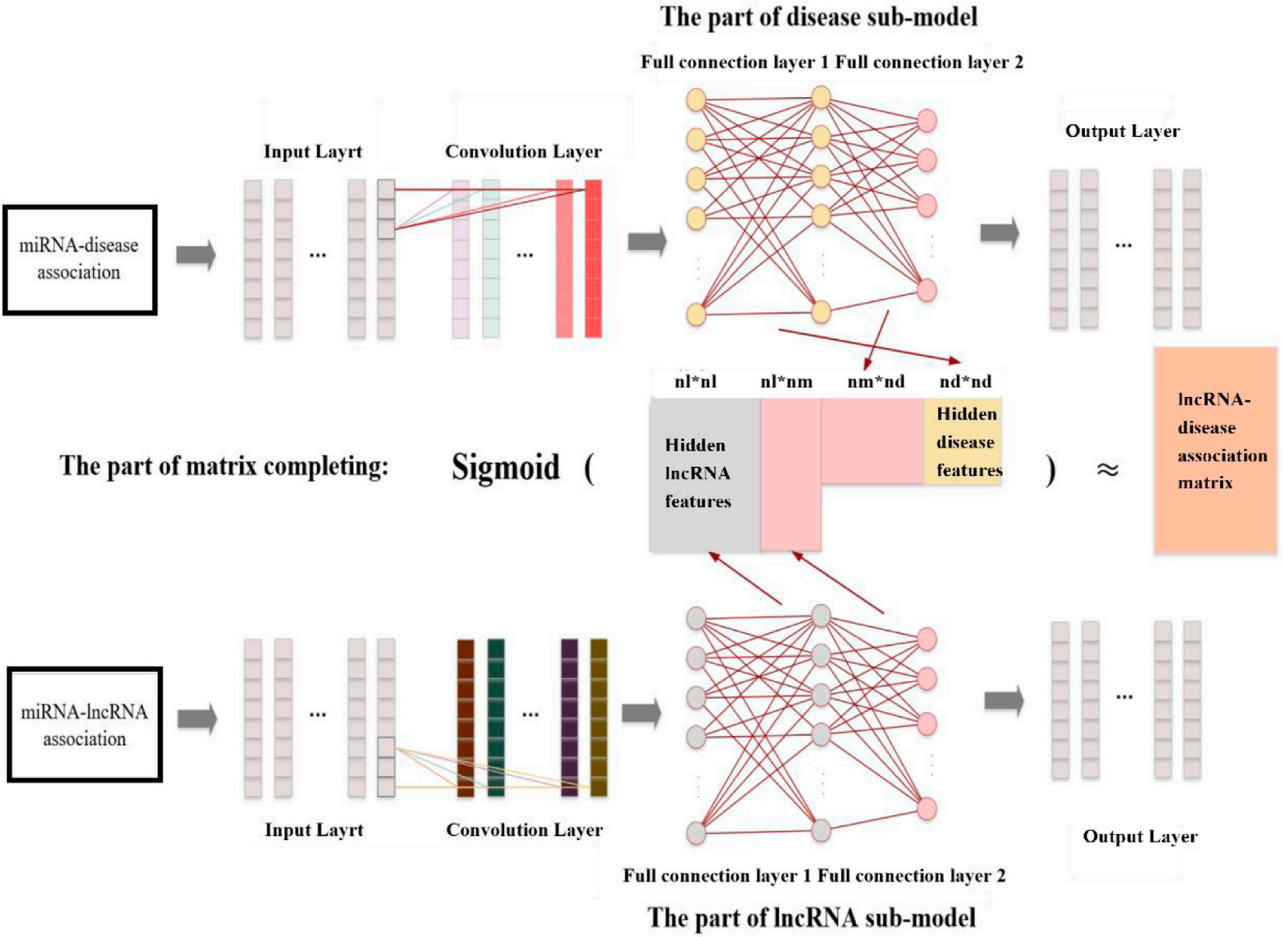
Flowchart of CNMCLDA.

Different from above traditional method, CNMCLDA introduced a novel learning framework to fill in the matrix *LD*. As shown in Figure 1, CNMCLDA consists of three major parts including the disease sub-model part, the lncRNA sub-model part and the part of matrix completion. Among them, the disease sub-model part and the lncRNA sub-model part are utilized to extract hidden features of diseases and lncRNAs by adopting CNNs separately, while the part of matrix completion is designed to obtain the predicted scores of possible lncRNA-disease associations.

Specifically, the main processes of CNMCLDA can be described as follows:

Step 1 : Designing a CNN for the disease sub-model part to extract hidden features of diseases by inputting *MD*.
Step 2 : Designing a CNN for the lncRNA sub-model part to extract hidden features of lncRNAs by inputting *ML*.
Step 3 : Calculating the predicted score matrix of lncRNA-disease associations based on the newly obtained hidden features of diseases and lncRNAs.
Step 3.1: Balancing positive and negative samples in *LD*.
Step 3.2: Constructing loss functions for CNNs.
Step 3.3: Calculating error values based on loss functions and updating parameters in CNMCLDA by back propagation.
Step 4 : Repeating steps 1 through 3 until CNMCLDA reaches a steady state.

### 3.1 Design of CNN for the disease sub-model to extract hidden features of diseases

CNN is a common deep learning architecture that excels in image recognition, natural language processing, etc (Gu et al., 2018). In this section, we would design a CNN consisting of a convolutional layer and three fully-connected layers for the disease-sub model part to extract hidden features of diseases firstly. For convenience, we would set the number of convolutional kernels to *nd*, and let $W_{d}^{i}$ and $B_{d}^{i}$ denote the weight matrix and biases in the *i*th layers of the CNN separately, then for each input *MD*(*i*), which represents the *i*th column of the matrix *MD*, its *j*th feature map can be calculated as the Formula 2:

$${fd}_{j}=f\left(MD\left(i\right)⨂W_{d}^{1}\left(j\right)+B_{d}^{1}\left(j\right)\right)$$ (2)

where ⨂ denotes convolution operations, $W_{d}^{1}\left(j\right)$ represents the weight matrix corresponding to the *j*th convolutional kernel, and *f*(*x*) stands for the activation function. There are some common activation functions, including Sigmoid, Tanh, ReLU, etc. Considering the efficiency and some possible problems (gradient disappearance and gradient explosion, etc.), we chose the ReLU as the activation function for CNMCLDA, which is defined as the Formula 3:

$$ReLU\left(x\right)=\max\left(0,x\right)$$ (3)

Thereafter, we can integrate all these feature maps as outputs of the convolutional layer.

Additionally, in these three fully-connected layers, the inputs of each layer can be derived by combining outputs of the previous layer with the weight matrix $W_{d}^{i}$ and biases $B_{d}^{i}$ , and then, the output of this layer can be obtained through the activation function. The dimension of the weight matrix $W_{d}^{i}$ can be set as the Formula 4:

$$size\;of\;W_{d}^{i}=\left\{\begin{array}{c} nd\times nd\;if\;i=2\\ nd\times nm\;if\;i=3\\ nm\times 1\;if\;i=4 \end{array}\right.$$ (4)

### 3.2 Design of CNN for the lncRNA sub-model to extract hidden features of lncRNAs

In this section, we would further design a CNN consisting of a convolutional layer and three fully-connected layers for the lncRNA sub-model part to extract hidden features of lncRNAs. In a similar way, For convenience, we would the number of convolutional kernels to *nl*, and let $W_{l}^{i}$ and $B_{l}^{i}$ denote the weight matrix and biases in the *i*th layers of CNN respectively, then for each input *ML*(*i*), which represents the *i*th column of the matrix of *ML*, its *j*th feature map can be calculated as the Formula 5:

$${fl}_{j}=f\left(ML\left(i\right)⨂W_{l}^{1}\left(j\right)+B_{l}^{1}\left(j\right)\right)$$ (5)

Thereafter, by combining all these feature maps, we can obtain the output of the convolutional layer as well. And moreover, the dimension of the weight matrix $W_{l}^{i}$ can be set as the Formula 6:

$$size\;of\;W_{l}^{i}=\left\{\begin{array}{c} nl\times nl\;if\;i=2\\ nl\times nm\;if\;i=3\\ nm\times 1\;if\;i=4 \end{array}\right.$$ (6)

### 3.3 Calculating the predicted score matrix of lncRNA-disease associations

Firstly, as the number of known lncrNa-disease associations is very limited, the number of elements equal to 0 in the original lncrNa-disease association matrix *LD* is far greater than the number equal to 1. For convenience, we call these elements equal to 0 or 1 as negative samples and positive samples, respectively, it is obvious that the proportion of positive samples and negative samples in the original lncrNa-disease relationship matrix *LD* is quite unbalanced, which makes it unreasonable to directly implement CNMCLDA on the original lncrNa-disease relationship matrix *LD*. Therefore, before implementing CNMCLDA, we will implement a division on the positive and negative samples of *LD* to ensure the approximate balance of positive and negative samples. Inspired by the method of KATZ (Sun et al., 2014), we will first construct a matrix *FLD* as the Formula 7:

$$FLD=LD\;*\;{LD}^{T}\;*\;LD+LD\;*\;S_{D}\;*\;S_{D}+S_{L}\;*\;S_{L}\;*\;LD+S_{L}\;*\;LD\;*\;S_{D}$$ (7)

And then, we will randomly select negative samples with amount equaling to the number of positive samples from the part of the matrix *FLD* with element of 0. Obviously, in this way, the positive and negative samples will be approximately balanced.

Next, considering that our main objective is to fill in the missing values in *LD*, therefore, based on features extracted from two CNNs, we will define the main loss function as the Formulas 8, 9:

$${loss}_{1}={\left\|LD-Sigmoid\left(W_{l}^{2}\;*\;W_{l}^{3}\;*\;W_{d}^{3T}\;*\;W_{d}^{2T}\right)\right\|}_{F}^{2}$$ (8)

$$Sigmoid\left(x\right)=\frac{1}{1+e^{-x}}$$ (9)

where ${\left\|A\right\|}_{F}^{2}$ denotes the Frobenius norm of the matrix *A*.

Obviously, the above Formula 8 can only be used to calculate the error values of heterogeneous nodes (i.e., the positive and negative samples in *LD*) in the sample set.

However, in CNNs of the disease-sub model and lncRNA sub-model, since we hope that the output of each CNN will be equivalent to the associations between the current node and all miRNA nodes, hence, we will define another loss function for this purpose as Formula 10:

$${loss}_{2}=\sum_{i}^{{set}_{d}}{\left\|{output}_{d}\left(i\right)\left.\;-\;MD\left(i\right)\right\|\right.}_{F}^{2}+\sum_{i}^{{set}_{l}}{\left\|{output}_{l}\left(i\right)\left.\;-\;ML\left(i\right)\right\|\right.}_{F}^{2}$$ (10)

where ${output}_{d}\left(i\right)$ and ${output}_{l}\left(i\right)$ represent the output of layer *i* of CNN in the disease-sub model and lncRNA sub-model, respectively.

Moreover, by combining the semantic similarity of disease with the functional similarity of lncRNA, we can define a novel loss function as Formula 11:

$${loss}_{3}={\left\|W_{l}^{2}-S_{l}\right\|}_{F}^{2}+{\left\|W_{d}^{2}-S_{d}\right\|}_{F}^{2}$$ (11)

Additionally, based on the framework of general matrix completion model, we can define a novel loss function as Formula 12:

$${loss}_{4}={\left\|W_{l}^{2}\right\|}_{F}^{2}+{\left\|W_{l}^{3}\right\|}_{F}^{2}+{\left\|W_{d}^{2}\right\|}_{F}^{2}+{\left\|W_{d}^{3}\right\|}_{F}^{2}$$ (12)

Finally, based on the weights and bias in the CNN, we can further define a novel loss function as Formula 13:

$${loss}_{5}=\sum_{i}^{\left\{l,d\right\}}\sum_{j}^{\left\{1,2,3,4\right\}}{\left\|W_{i}^{j}\right\|}_{F}^{2}+{\left\|B_{i}^{j}\right\|}_{F}^{2}$$ (13)

Thereafter, by integrating above five loss functions, we can obtain a total loss function as Formula 14:

$${loss}_{total}={loss}_{1}+{\lambda_{1}\;*\;loss}_{2}+{\lambda_{2}\;*\;loss}_{3}+{\lambda_{3}\;*\;loss}_{4}+\lambda_{4}\;*\;{loss}_{5}$$ (14)

Finally, based on the total loss function, we can further adopt the Adam optimization method (Shi et al., 2024) to iteratively optimize the hyper-parameters in CNMCLDA. And In the actual deployment CNMCLDA, considering the time cost and precision requirements, the iteration process will stop when the value of *loss*_*total*_ is less than 10^−3^. Hence, we can finally obtain the predicted scores of possible lncRNA-disease associations as Formula 15:

$$LD^{\prime }=Sigmoid\left(W_{l}^{2}\;*\;W_{l}^{3}\;*\;W_{d}^{3T}\;*\;W_{d}^{2T}\right)$$ (15)

## 4 Performance evaluation

In this section, we compared CNMCLDA with seven state-of-the-art models including NBCLDA (Jingwen et al., 2018), CFNBC (Yu et al., 2019), PMFILDA (Xuan et al., 2019), gGATLDA (Wang and Zhong, 2022), LDAGRL (Zhang et al., 2023), IIRWR (Wang et al., 2019), FVTLDA (Xiao et al., 2020), BIWALK (Hu et al., 2019), and LRWHLDA (Li et al., 2021). Among these competitive models, IIRWR, BIWALK and LRWHLDA adopt network propagation-based methods to infer potential lncRNA-disease associations, while NBCLDA, CFNBC, PMFILDA, gGATLDA, LDAGRL and FVTLDA adopt machine learning-based methods to predict potential associations between lncRNAs and diseases.

During experiments, frameworks of *K*-fold CV including 5-fold CV and 10-fold CV would be employed first to compare the prediction performances between CNMCLDA and all these competing models. While implementing *K*-fold CV, known lncRNA-disease associations would be divided into *K* equal subsets randomly, and each subset was left out as the test sample, whereas the remaining *K*-1 subsets were retained as training samples (Zhou et al., 2018). Moreover, all test samples and unknown lncRNA-disease associations would be considered as candidate samples. Hence, after ranking these candidate samples according to their predicted scores obtained by experiments, for a given threshold, we could obtain the *TPR* (True Positive Rate) and *FPR* (False Positive Rate) of each method according to the following Formulas 16, 17 separately:

$$TPR=\frac{TP}{TP+FN}$$ (16)

$$FPR=\frac{FP}{FP+TN}$$ (17)

where *TP* (True Positive) and *FP* (False Positive) represent the numbers of known and unknown lncRNA-disease associations with scores above the given threshold respectively, while *FN* (False Negative) and *TN* (True Negative) denote the numbers of known and unknown lncRNA-disease associations with scores below the given threshold respectively.

**FIGURE 2:**
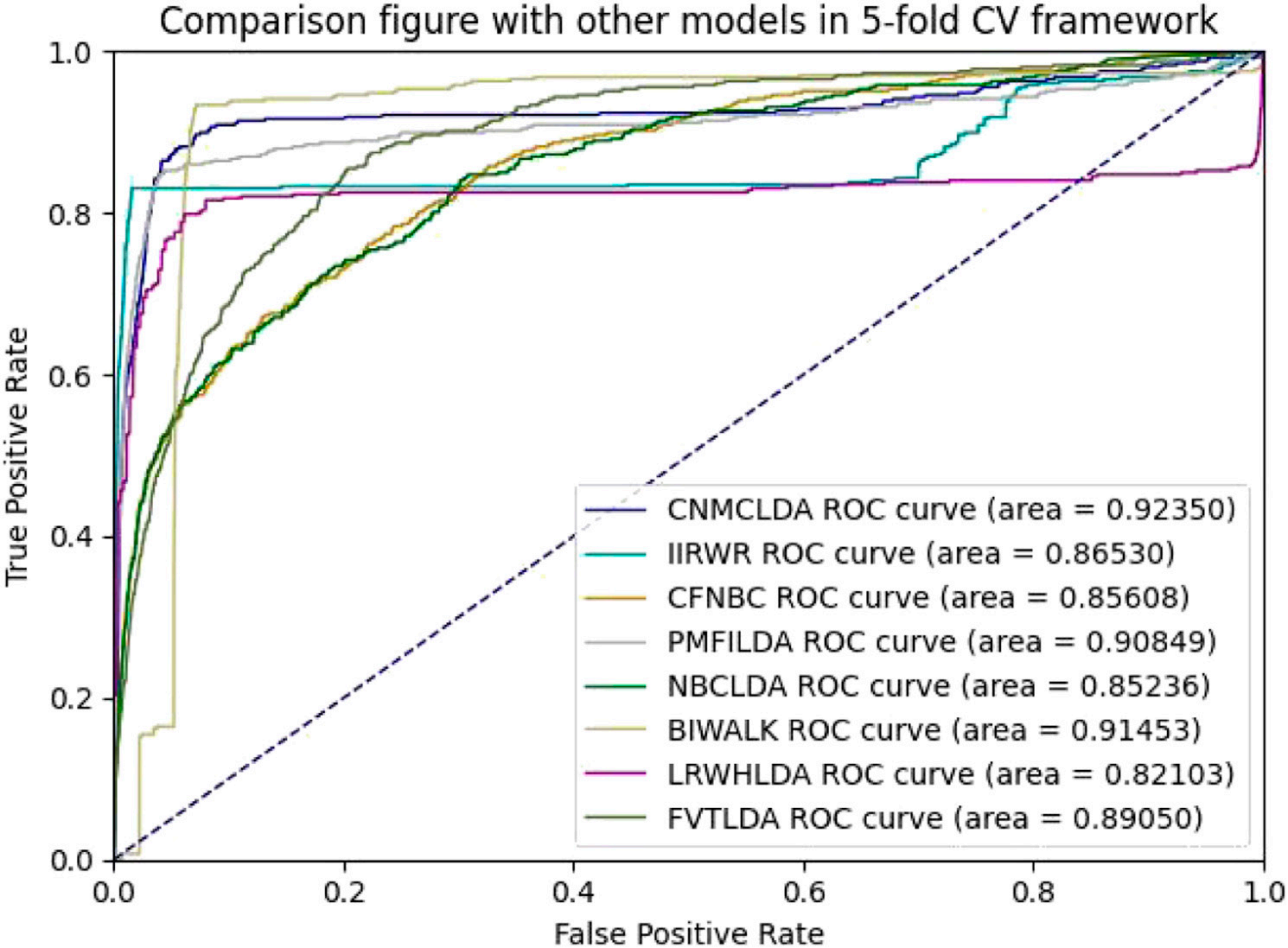
The AUCs achieved by CNMCLAD, IIRWR, PMFILDA, NBCLDA, CFNBC, BIWALK, LRWHLDA and FVTLDA in framework of five-fold CV.

**FIGURE 3:**
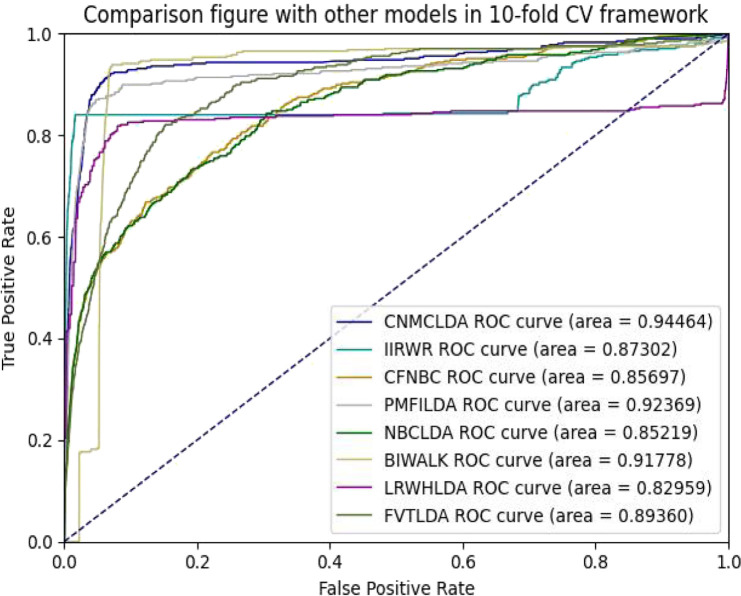
The AUCs achieved by CNMCLAD, IIRWR, PMFILDA, NBCLDA, CFNBC, BIWALK, LRWHLDA and FVTLDA in framework of ten-fold CV.

Obviously, through setting different thresholds, a unique ROC (Receiver operating characteristic) curve could be obtained by plotting *TPRs* against *FPRs* for each method. Thereafter, the AUC (Area Under the ROC Curve) could be used to evaluate the prediction performance of the given method (Hand and Till, 2001). As shown in Figures 2, 3, while implementing the 5-fold CV, CNMCLDA achieved reliable AUC value of 0.92350, which was significantly higher than all competitive models such as IIRWR (0.8653), CFNBC (0.85608), PMFILDA (0.90849), NBCLDA (0.85236), BIWALK (0.91453), LRWHLDA (0.82103), gGATLDA (0.92216), LDAGRL (0.92158), and FVTLDA (0.89050). While implementing the 10-fold CV, CNMCLDA achieved reliable AUC value of 0.94464, which was much better than all competitive models such as IIRWR (0.87302), CFNBC (0.85697), PMFILDA (0.92369), NBCLDA (0.85219), BIWALK (0.91778), LRWHLDA (0.82959), gGATLDA (0.94420), LDAGRL (0.93162), and FVTLDA (0.89360) as well.

**FIGURE 4:**
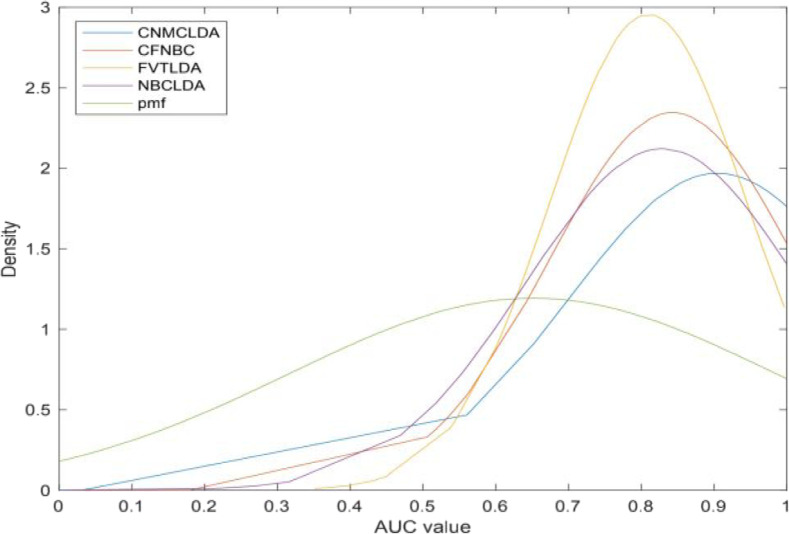
Performance comparison of CNMCLDA with the other four computational models in framework of LODOCV.

Moreover, in order to further evaluate the predictive ability of CNMCLDA, we introduced a novel evaluation metric called LODOCV, which could be implemented as follows: for a given disease *d*, all lncRNAs having known associations with *d* would be left out as test samples, while the remaining lncRNAs were utilized for prediction. Especially, considering that IIRWR, BIWALK and LRWHLDA are RW (Random Walk)-based methods, which cannot be used to predict lncRNAs that have no known associations with any disease, we compared CNMCLDA only with the remaining four predictive models such as CFNBC, FVTLDA, NBCLDA and PMFILDA. As shown in Figure 4, CNMCLDA achieved much better predictive performance than all these four competitive models. And meanwhile, in order to show the prediction performance of CNMCLDA more intuitively, we illustrated the means and variances of AUCs of CNMCLDA and all these four competitive models in Table 2, and the statistical significance of the predictive performance difference between CNMCLDA and all these four competitive models in Table 3, respectively.

**TABLE 2:** Comparison of the means and variances of AUCs between CNMCLDA and four competitive models in the framework of LODOCV.

Model	Mean of AUC values	Variance of AUC values
CNMCLDA	0.9041	0.0411
CFNBC	0.8427	0.0289
FVTLDA	0.8097	0.0182
NBCLDA	0.8289	0.0938
PMFILDA	0.6506	0.1118

**TABLE 3:** Comparison of the statistical significance of performance differences between CNMCLDA and four competitive models in the framework of LODOCV.

Model	CFNBC	FVTLDA	NBCLDA	PMFILDA
P-value	3.68E-08	7.30E-11	2.88E-08	1.66E-09

Finally, under the framework of LOOCV (Leave-One-Out Cross Validation), we further compared the AUCs achieved by CNMCLDA and HGLDA (Chen, 2015b) based on the dataset proposed by HGLDA, which consists of 183 known lncRNA-disease associations that have been confirmed by experiments. While implementing LOOCV, each known lncRNA-disease association are selected out in turn as the test sample, and the rest associations are regarded as the training samples (Bo et al., 2006). As illustrated in Table 4, CNMCLDA can achieve an AUC of 0.8546, which is much higher that the AUC of 0.7621 achieved by HGLDA.

**TABLE 4:** Comparison of prediction performances between CNMCLDA and HGLDA based on the dataset proposed by HGLDA.

Model	AUC value
CNMCLDA	0.8546
HGLDA	0.7621

Therefore, it can be seen from above descriptions that CNMCLDA can achieve better prediction performance than existing state-of-the-art models.

## 5 Parameter analysis

As described in the method section, there are four hyper-parameters in CNMCLDA. In this section, we would evaluate the impacts of these parameters on the predictive performance of CNMCLDA under the framework of 5-fold CV. During experiments, we estimated the performance of remaining 3 parameters by fixing one parameter, and the range of each parameter would be set to {0.001,0.01,0.1,1,10} respectively. Finally, we found that CNMCLDA could achieve the best predictive results (see Supplementary Material S1) while these parameters were set as follows:

$$\lambda_{1}=10^{-3},\lambda_{2}=10^{-1},\lambda_{3}=10,\lambda_{4}=1.$$

## 6 Case study

In this section, in order to demonstrate the effectiveness and practicability of CNMCLDA, we implemented case studies of gastric cancer, glioma, and breast cancer on known dataset having confirmed by experiments. During experiments of case studies, for a given disease *d*, we first regarded all lncRNAs having no known associations with *d* as candidates. Thereafter, all candidate lncRNAs would be ranked according to their prediction scores calculated by CNMCLDA. Finally, we would validate the relationships between top 20 candidate lncRNAs and *d* by the recently published papers in NCBI database (https://www.ncbi.nlm.nih.gov/).

Gastric cancer is the second most frequently leading cause of death in cancer, and it is also the fourth most common cancer in the word (Hartgrink et al., 2009; Guo et al., 2014). Recently, a larger number of literatures have confirmed the relationship between lncRNAs and gastric cancer, and lncRNAs may be therapeutic targets in patients with gastric cancer. For example, Chen et al. found that the upregulation of lncRNA XIST was related to aggressive tumor phenotypes and survive of gastric cancer (Chen et al., 2016). Yang et al. pointed out that the level of H19 in gastric cancer cells and tissues was significantly higher than that in normal control (Yang et al., 2012). As shown in Table 5(a), we listed top 20 candidate lncRNAs predicted by CNMCLDA, only one of these top 20 candidate lncRNAs has not been confirmed by recent relevant literatures. Moreover, all remaining 19 lncRNAs having been verified to be related to gastric cancer were attached with corresponding PMID (PubMed unique identifiers) in Table 5(a) as well.

**TABLE 5:** Top 20 potential {gastric cancer, glioma, breast cancer}-related lncRNAs predicted by CNMCLDA and their PubMed unique identifiers.

(a). Gastric cancer		(b). Glioma		(c). Breast cancer	
lncRNA	Evidence (PMID)	lncRNA	Evidence (PMID)	lncRNA	Evidence (PMID)
XIST	27911852	CCAT1	28475287	TUG1	30098551
GAS5	31182630	XIST	28287613	CASC2	30106139
MALAT1	32104001	GAS5	31889362	HOTTIP	32307830
NEAT1	28401449	HOTAIR	28083786	TINCR	30621694
TUG1	29719612	MEG3	32271438	FENDRR	29559798
ZFAS1	Unknown	MALAT1	32117213	TP53TG1	Unknown
CASC2	30372881	NEAT1	30515782	LINC00473	30848493
KCNQ1OT1	31915311	TUG1	29467911	HOXA11-AS	28791375
HOTTIP	31908497	ZFAS1	31535380	HOTAIRM1	32284737
PVT1	31966056	CASC2	28121023	CRNDE	28469804
TINCR	28744139	KCNQ1OT1	28381990	MIAT	29100300
FENDRR	25167886	HOTTIP	28886531	ZNRD1-AS1	Unknown
DANCR	31002130	PVT1	31957841	SNHG16	32122142
TP53TG1	27821766	TINCR	Unknown	SNHG5	31255976
LINC00511	32042282	FENDRR	Unknown	TDRG1	Unknown
LINC00473	30071345	H19	31173296	HOXA-AS2	28545023
HOXA11-AS	32009419	DANCR	29940760	SNHG15	32021307
HOTAIRM1	30302796	CDKN2B-AS1	Unknown	SNHG7	31897328
CRNDE	28490034	TP53TG1	28569381	LINC00313	Unknown
TP73-AS1	30279010	LINC00511	30973678	LINC00152	30594392

As for glioma, it is a major type of adult intracranial tumors. High grade gliomas tend to infiltrate into brain extracellular matrix, which makes surgery and radiotherapy difficult (Gwak et al., 2012). A lot of evidences show that lncRNAs plays an important role in glioma. For instance, Wang et al. demonstrated that lncRNA CASC2 can play an anti-tumor role in glioma through negative regulation of MicroRNA-21 (Wang et al., 2015). Wang et al. pointed out that lncRNA HOXA11-AS is a biomarker to identify glioma and can be used as a therapeutic target for glioma patients (Wang et al., 2016). As illustrated in Table 5(b), we found 17 lncRNAs out of top 20 candidate lncRNAs predicted by CNMCLDA having been confirmed to be related to gastric cancer in recent relevant literatures.

Moreover, breast cancer is the most common cancer, and it is also the main cause of cancer death in women all over the world (DeSantis et al., 2015). Up to now, there are many relevant literatures demonstrating the relationship between lncRNA and breast cancer, such as lncRNA H19 (Sun et al., 2015), lncRNA UCA1 (Xiao et al., 2016), lncRNA HOTAIR (Xue et al., 2015) and so on. As illustrated in Table 5(c), there are 16 out of top 20 candidate lncRNAs predicted by CNMCLDA having been reported in recent literatures. Hence, based on experimental results of above case studies, we can conclude that CNMCLDA has excellent prediction ability.

## 7 Discussion

In this study, different from existing methods, we regarded the prediction of potential diseases-related lncRNAs as completion of missing values of the lncRNA-disease relational matrix, and defined a novel end-to-end learning framework CNMCLDA to infer potential lncRNA-disease associations. The main contribution of CNMCLDA includes: (1) lots of existing methods are strongly dependent on known lncRNA-disease associations, however, CNMCLDA combines a variety of biological information to ensure that it does not rely only on known lncRNA-disease associations, which makes it suitable for inferring potential associations between lncRNAs and isolated diseases. (2) traditional machine learning based methods randomly select unlabeled samples as negative samples, or directly take all unlabeled samples as negatives, while CNMCLDA takes into account the balance of positive and negative samples, enabling it to achieve better predictive performance. (3) five different loss functions are designed to optimize the parameters of CNMCLDA synchronously, which makes it more effective. Certainly, CNMCLDA still has rooms for improvement. For instance, the neural network can be designed more complicated by combining the symptoms and pathological stages of diseases, and multi-view learning can be carried out as well. Meanwhile, known lncRNA-disease associations can be divided into upregulation and downregulation parts for multi-label learning. Finally, how to balance the four trade-off parameters in loss functions to achieve global optimal solutions is still a challenging task. Moreover, One of the major characteristics of lncRNA is that it has good tissue specificity and cell type specificity (Grassi et al., 2021), so it is very suitable for the study of specific related mechanisms. In terms of tumors, inflammation, immune diseases, neurological diseases, lncRNA provides a good tool for the study of heterogeneity, and is particularly suitable for future studies in marker mining or target discovery.

## Data Availability

The original contributions presented in the study are included in the article/Supplementary Material, further inquiries can be directed to the corresponding authors.
